# Harnessing Immune Response in Acute Myeloid Leukemia

**DOI:** 10.3390/jcm12185824

**Published:** 2023-09-07

**Authors:** Carola Riva, Chiara Vernarecci, Paola Minetto, Rayan Goda, Marco Greppi, Silvia Pesce, Maria Chies, Giada Zecchetti, Beatrice Ferro, Elena Maio, Michele Cea, Roberto Massimo Lemoli, Emanuela Marcenaro, Fabio Guolo

**Affiliations:** 1 Clinic of Hematology, Department of Internal Medicine, University of Genova, 16132 Genova, Italy; carola.riva@outlook.it (C.R.); chiaravernarecci@yahoo.it (C.V.); maria.chies96@gmail.com (M.C.); giadazecchetti@gmail.com (G.Z.); ferrobeatrice1@gmail.com (B.F.); elenamaio1996@gmail.com (E.M.); michele.cea@unige.it (M.C.); roberto.lemoli@unige.it (R.M.L.); fabio.guolo@unige.it (F.G.); 2 IRCCS Ospedale Policlinico San Martino, 16132 Genova, Italy; emanuela.marcenaro@unige.it; 3 Department of Experimental Medicine (DIMES), University of Genova, 16132 Genova, Italy; rayangoda@gmail.com (R.G.); marco.greppi@edu.unige.it (M.G.); silvia.pesce@unige.it (S.P.)

**Keywords:** AML, immunotherapy, CAR-T, NK cells

## Abstract

Despite the results achieved with the evolution of conventional chemotherapy and the inclusion of targeted therapies in the treatment of acute myeloid leukemia (AML), survival is still not satisfying, in particular in the setting of relapsed/refractory (R/R) disease or elderly/unfit patients. Among the most innovative therapeutic options, cellular therapy has shown great results in different hematological malignancies such as acute lymphoblastic leukemia and lymphomas, with several products already approved for clinical use. However, despite the great interest in also expanding the application of these new treatments to R/R AML, no product has been approved yet for clinical application. Furthermore, cellular therapy could indeed represent a powerful tool and an appealing alternative to allogeneic hematopoietic stem cell transplantation for ineligible patients. In this review, we aim to provide an overview of the most recent clinical research exploring the effectiveness of cellular therapy in AML, moving from consolidated approaches such as post- transplant donor’s lymphocytes infusion, to modern adoptive immunotherapies such as alloreactive NK cell infusions, engineered T and NK cells (CAR-T, CAR-NK) and novel platforms of T and NK cells engaging (i.e., BiTEs, DARTs and ANKET^TM^).

## 1. Introduction

Acute myeloid leukemia (AML) is a clonal disease characterized by a broad spectrum of cytogenetic and molecular abnormalities that influence clinical outcomes.

AML treatment for young and fit patients relies on intensive anthracyclines and cytarabine-based chemotherapy followed by allogenic hematopoietic stem cell transplantation (allo-HSCT) in selected patients. Treatment options in AML have recently expanded with the introduction of immune-conjugates, such as gemtuzumab-ozogamicin (GO) or tagraxofusp, and new targeted molecules; for example FLT3, IDH1, IDH2 and BCL2 inhibitors [1]. However, the survival rate is still unsatisfying, especially in the setting of relapsed/refractory (R/R) disease or elderly/unfit patients.

Allo-HSCT consolidation is recommended for high-risk patients (i.e., those harboring adverse cytogenetic and molecular aberrations or those with positive minimal residual disease) in order to reduce the risk of relapse and prolong survival [2]. The anti-leukemic effect of allo-HSCT mainly relies on the immunological cytolysis of residual leukemic cells mediated by the adoptively transferred donor’s immune cells (graft-versus-leukemia, GvL). In view of this, allo-HSCT can be considered the first successful clinical application of cellular immunotherapy [3]. However, allo-HSCT is aggravated by the significant toxicities associated with the conditioning regimen, and by the risk of graft-versus-host disease (GvHD), therefore limiting the eligibility to allo-HSCT to only younger and fit patients. Recently, adoptive cellular therapy (i.e., CART-T cells) has shown considerable results in different hematological malignancies such as acute lymphoblastic leukemia and lymphomas, with several products already approved for clinical use [4]. Despite the great interest in expanding the application of these new treatments to R/R AML, no product has been approved yet for this indication. This is partly due to the lack of a “pan-AML”-specific surface target, since many leukemia-associated antigens are commonly expressed on healthy myeloid cells, resulting in on-target-off-leukemia myeloablative effects [5].

In this view, novel therapeutic approaches based on cell-based and adoptive immunotherapy represent an appealing strategy to overcome chemoresistance (i.e., in R/R or MRD positive patients) and alternative, less toxic consolidation strategies for patients ineligible for standard allo-HSCT. 

This review will provide updates on the most recent clinical research exploring the effectiveness of cellular therapy in AML, moving from consolidated approaches such as post-transplant donor’s lymphocytes infusion, to modern adoptive immunotherapies such as alloreactive Natural Killer (NK) cell infusions, engineered T and NK cells (CAR-T, CAR-NK) and novel platforms of T and NK cells engagement (i.e., BiTEs, DARTs and ANKET_TM_).

## 2. T and NK Cells

**Figure 1:**
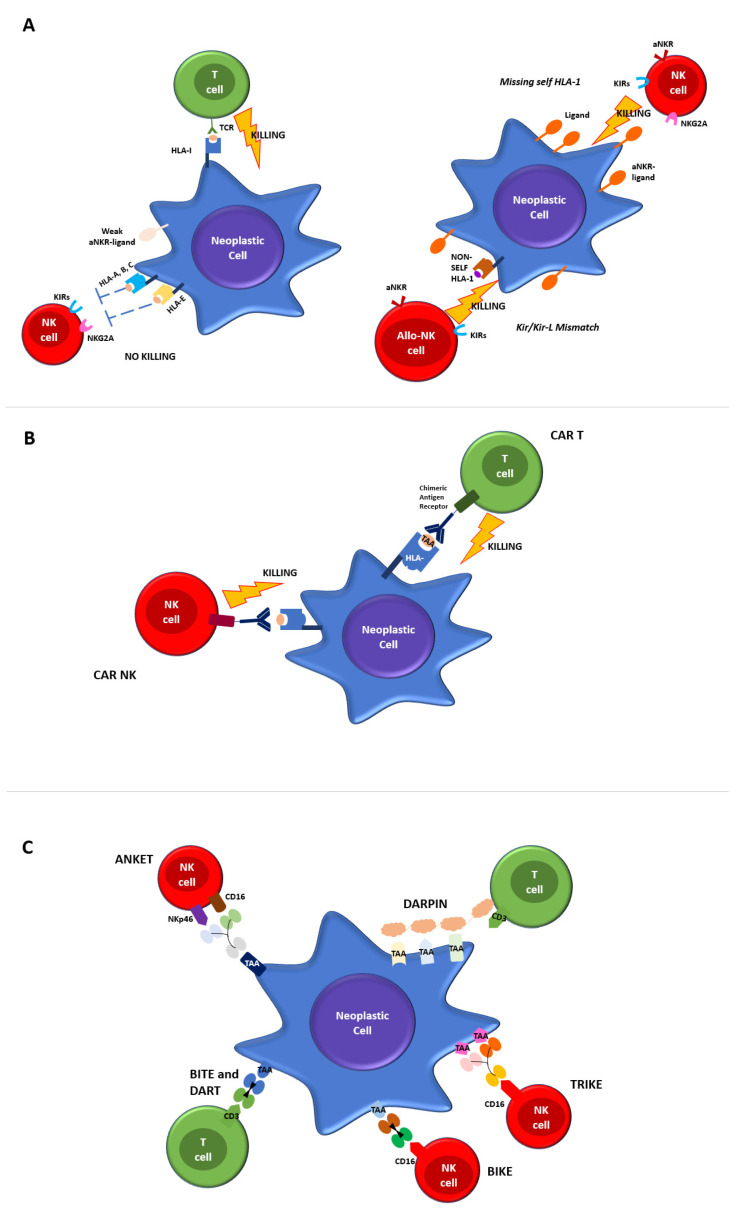
Mechanisms of T and NK-cell mediated killing. (**A**) Regulation of T and NK killing in physiological conditions. Cytotoxic T Lymphocytes express antigen-specific T cell receptors (TCRs) to recognize cognate peptides (antigens) bound to human leukocyte antigen (HLA) molecules on target cells. The binding of peptide/HLA-I complexes by TCR initiates T cell activation that converges in cytolytic activity. NK cell mediated-killing is regulated by complex interactions between inhibitory and activating receptors binding various ligands, including HLA-I, that prevents the killing of healthy autologous cells expressing appropriate levels of all self-HLA alleles and low/negative levels of ligands for activating receptors (left panel). The downregulation of HLA-I molecules on neoplastic cells induces NK-mediated killing by a “missing-self” recognition mechanism. NK cell-activating receptors are co-responsible for NK activation interacting with ligands overexpressed on neoplastic cells. Furthermore, NK cells kill neoplastic cells through the recognition of non-self HLA-I molecules (“KIR/KIR-ligand mismatch”), a mechanism of immunosurveillance active in the context of allogeneic-hematopoietic stem cell transplantation and in strategies of adoptive immunotherapies by alloreactive NK cells infusion (right panel). (**B**) CTLs and NK engineered with chimeric antigen receptors (CAR). The chimeric antigen receptor is made by a monoclonal antibody capable of binding to a specific identified target antigen, coupled with the activation of the intracellular proliferation signal domain. When the chimeric receptor binds to the antigen expressed in malignant cells, it stimulates the activation signal, leading to CAR-T cell activation and to the killing effects. (**C**) Novel platforms for T and NK cells engaging (see text for details).

The immune system plays an essential role in fighting against infections, inflammation, autoimmunity and cancer. Immune cells exert control in the early stages of cancer development, through the killing of malignant cells, thus hampering the chance of further tumor growth. The main immune cells involved in anti-tumor responses are cytotoxic CD8+ T cells (CTL) and Natural Killer (NK) cells, which are components of the adaptive and innate immune system, respectively (Figure 1A). 

CTLs express antigen-specific T cell receptors (TCRs) to recognize cognate peptides (antigens) bound to human leukocyte antigen (HLA) molecules on target cells. The binding of peptide/HLA-I complexes by TCR initiates T cell activation that converges in cytolytic activity.

In contrast, NK cells lack specific antigen receptors but express a repertoire of inhibitory and activating receptors that bind various ligands, including HLA-I. A highly calibrated “education process” during the different steps of NK cells maturation allows the elimination of target cells with reduced or absent HLA-I molecules (“missing self”) while maintaining their tolerance towards cells expressing adequate levels of self-HLA. The main inhibitory NK receptors, KIRs and CD94/NKG2A, recognize classical (HLA-ABC) and non-classical (HLA-E) HLA-I molecules, respectively. 

Activating NK receptors include a variety of non-HLA-specific receptors and co-receptors, which trigger NK cell stimulation via a straight interaction with ligands over/neo-expressed on malignant or virus-infected cells [6].

Both CTL and NK cells, upon activation, undergo membrane reorganization and express various effector molecules to eliminate aberrant cells subjected to tumor transformation or infection by intracellular pathogens. Figure 1A provides a detailed overview of physiological T and NK cell-mediated killing.

T and NK cells mediated tumor killing occurs via direct cell cytotoxicity, the release of perforin and granzyme, NK cells antibody-dependent cellular cytotoxicity (ADCC) via engaging their receptor (CD16) or by apoptotic axis intermediated by the Fas ligand (FasL) or the TNF-related apoptosis-inducing ligand (TRAIL). T and NK cells-mediated anti-tumor immune responses lead to the secretion of pro-inflammatory cytokines, including interferon-γ (IFN-γ), and chemokines. 

Several mechanisms of immune escape have been identified and are associated with AML development and relapse. In addition to being poorly immunogenic, AML blasts are able to evade immune effector cells via the downregulation of antigen presentation machinery (MHC class I and class II) resulting in CD8+ T cell tolerance [6,7,8,9].

Indeed, a complex interplay between AML cells, bone marrow (BM) microenvironment and immune effector cells result in the dysregulation of innate and adaptive immune responses. A pivotal role in the induction of immune-tolerance with the enrichment of T regulatory cells in the context of the BM microenvironment is played by mesenchymal stromal cells, which support leukemic blasts and create a protective environment in response to pro-inflammatory stimuli [10,11].

AML cells have also been shown to induce exhaustion in activated T cells via modulating the expression of immune-checkpoint receptors, such as programmed cell death protein-1 (PD-1), cytotoxic T lymphocyte antigen-4 (CTLA-4), T-cell immunoglobulin and mucin-domain containing-3 (TIM-3) and lymphocyte-activation gene 3 (LAG3). Furthermore, functionally impaired NK cells characterized by the upregulation of inhibitory KIRs have been identified in AML patients at diagnosis [3].

**Figure 2:**
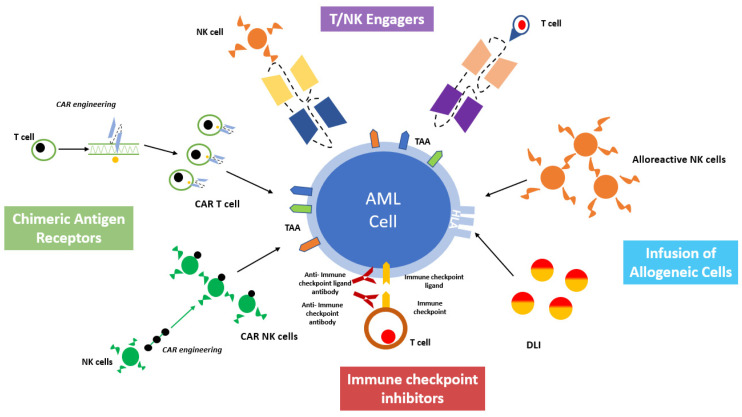
A comprehensive overview of cell-based immunotherapy strategies for acute myeloid leukemia treatment.

A deeper understanding of the mechanisms regulating T and NK cell-mediated killing and AML interaction with the BM microenvironment paved the way for the development of effective cancer immunotherapies and post-transplant immunological interventions [12,13]. The major aim of these approaches is to harness the immune system against AML, both by eliciting the cytotoxic effectors or by inhibiting the tolerogenic milieu of the BM microenvironment. A comprehensive overview of cell-based immunotherapy is provided in Figure 2.

## 3. Principles of Adoptive Immunotherapy in Allogeneic Stem Cell Transplantation

### 3.1. Graft versus Leukemia Effect

Graft versus Leukemia (GvL) is defined as the anti-leukemic response mediated by donor’s immune cells in the context of allo-HSCT and has been the first established form of adoptive immunotherapy in cancer treatment. Donor’s T cells, which recognize residual leukemic cells through interactions with major histocompatibility (MHC) molecules on the surface of AML cells, are the major effector of adoptive immune-responses in allo-HSCT. The demonstration of a pivotal role of T cells mediating the GvL effect comes from past evidence of increased relapse rates observed using early T cell depleted graft platforms, as well as from the proven efficacy of post-transplant donor’s lymphocyte infusions (DLIs) in achieving disease control as both a prophylactic or pre-emptive strategy [14].

However, the GvL effect is not limited to T cell activity. The anti-leukemic function of donor-derived alloreactive NK cells has been also specifically elucidated in the context of T-depleted haplo-identical HSCT (haplo-HSCT), a platform based on graft manipulation with a negative depletion of αβ-T and B cells, which allows the infusion of mature NK and γδ-T cells together with high doses of donor’s HSC [15,16]. In this setting, early reports from Ruggeri et al. showed that KIR ligand incompatibility in the graft-versus-host (GvH) direction (KIR-ligand mismatch) was an independent predictor of reduced relapse risk in a cohort of high-risk acute leukemia patients receiving haplo-HSCT [17]. Further evidences confirmed improved disease-free survival (DFS) and reduced rates of leukemia relapse if a KIR-ligand mismatch was present as well as the persistence of alloreactive NK clones for a few years after HSCT, suggesting a mechanism of selection and in vivo expansion [18,19]. Moreover, alloreactive mismatched NK cells have been shown to facilitate hematopoietic engraftment after haplo-HSCT and inhibit the onset of GVHD by targeting host antigen-presenting cells [17,18,19,20,21]. Indeed, the receptor-mediated tight regulation of NK cell activity and the different expression of activating ligands on hematopoietic and not hematopoietic tissues may provide an explanation for the observed NK-mediated GvL effect in the absence of GvHD. This has led to the development of allo-reactive NK cells, engineered to express inhibitory KIRs that do not engage any of the HLA-I alleles present on allogeneic target cells, hence potentiating their ability to kill leukemic cells as well as host dendritic cells (DCs) and T cells. This approach significantly improved patient outcome post haplo-HSCT by directly preventing GvHD and Host versus Graft disease (HvGD) [22].

### 3.2. Donor’s Lymphocytes Infusion

Relapse after allo-HSCT still represents a major clinical challenge. Among mechanisms involved in leukemia relapse, CD4+/CD8+ T-cell exhaustion and the upregulation of inhibitor receptors (PD1 and Tim-3) on T-lymphocytes have been documented [23]. Overall, in AML patients undergoing allo-HSCT, post-transplant prophylactic or pre-emptive donor lymphocyte infusions (DLIs) represent a simple and valuable therapeutic strategy to reduce the incidence of leukemia relapse, whereas its efficacy is limited in patients in overt relapse. DLIs act by repleting donor T cell pools and potentially reversing T cell exhaustion through an increase in terms of the function and proliferation of the CD8+ T cell resident in the recipient bone marrow [24,25].

The main toxicity associated with DLIs is the triggering of GvHD, which is strictly related to the dose of T cell infused. The risk of GvHD is significantly reduced without hampering efficacy when multiple, escalating doses of DLI, rather than single high-dose infusions, are administered [26].

The first evidence of the anti-leukemic activity of DLIs was observed in Chronic Myeloid Leukemia (CML), in 1990 [27]; several groups demonstrated that DLIs were able to induce complete hematologic and cytogenetic remission in up to 80% of chronic-phase CML patients relapsing after allo-HSCT [28,29]. However, results in blast-phase CML or AML patients relapsing after allo-HSCT were significantly inferior, with less than 25% of patients achieving short-term remissions. In order to improve those results, earlier infusions of DLI, such as prophylactic (pro-DLI) or pre-emptive (pre-DLI) administration, were tested, based on the idea that immunologic intervention is likely to be more effective when performed on a lower tumor burden [30].

Minimal residual disease (MRD) or donor chimerism assessment can be used in order to identify AML patients in morphological complete remission (CR) at high risk of impending relapse, who therefore may benefit from pre-DLI administration.

AML patients with recurrent genetic abnormalities such as NPM1 mutation, Core Binding Factor AML or acute promyelocitic leukemia (APL) are the best candidates for such an approach, given the availability of highly sensitive MRD markers; several studies have shown that the administration of pre-DLI improves disease outcomes [31,32].

However, since most AML patients lack an AML-specific MRD marker, multicolor flow cytometry (MFC) MRD is a good strategy to predict relapse and to guide a pre-emptive intervention [33,34]. Tan et al. observed the efficacy of pre-DLI on 15 AML and ALL patients based on the detection of leukemia-associated immunophenotypic patterns (LAIPs) through a four-color MFC. They also demonstrated the superiority of pre-DLI compared to DLI infused after overt hematological relapse [35].

In a Chinese prospective trial, the administration of pre-DLI driven by MFC or Wilms Tumour gene (WT1) expression levels of MRD positivity was associated with a 2.3-fold reduction in relapse risk [36]. In a retrospective study, Dominietto et al. reported on the efficacy of a WT1-MRD-driven pre-DLI administration. The relapse rate was significantly decreased in patients who received pre-DLI compared to those who did not receive the immunological intervention [37].

Alternatively, donor chimerism analysis, which is routinely performed in allo-HSCT patients, can be used, although it is less sensitive compared to MRD techniques. Furthermore, the interval between the detection of a decrease in the percentage of donor cells and the overt relapse is often too short to enable successful pre-emptive therapeutic interventions [38,39].

Prophylactic DLI administration may be planned in patients without any evidence of disease reoccurrence but who are considered to be at high risk for relapse. In a matched-paired analysis from the European Society for Blood and Marrow Transplantation (EBMT) Acute Leukaemia Working Party (ALWP) registry, Schmid et al. evaluated the efficacy of pro-DLI in 105 AML and ALL patients, compared to 89 patients with matched disease specification who did not receive pro-DLI as a control population. Pro-DLI was administered within a year from SCT and with a median of two infusions. In patients with a standard risk of disease, no advantage was observed, while improved 5-year OS was observed in the high-risk group (69.8% vs. 40.2%, *p* = 0.027) [40].

To increase the efficacy of DLIs, a combination with drugs aimed at reducing the disease burden has been explored. The pyrimidine nucleoside analog 5-Azacytidine (AZA) has been shown to potentially increase DLI activity. The observed improvement in responses seems to be mainly related to an immunomodulatory effect of AZA, such as the upregulation of HLA expression and cancer-associated antigens [41].

## 4. Immune Checkpoint Inhibitors (ICI) as Immunotherapeutic Strategies for AML

Immune Checkpoints (IC) are proteins physiologically expressed on immune effector cells, acting as key regulators of T- and NK-mediated responses promoting self-tolerance. The discovery that cancer cells can mimic the IC ligands as a mechanism of immune escape led to the development of novel antitumor strategies with IC inhibitors (ICIs), which have become fundamental for the treatment of several solid organ malignancies [42]. The only approved indication for treatment with ICIs of hematologic malignancies is R/R Hodgkin’s Lymphoma [43].

In the AML setting, several ICIs have been investigated. The first ICI tested in AML was the CTLA-4 inhibitor Ipilimumab. It showed promising results in terms of the infiltration and expansion of effector T cells and reduced the activation of regulatory T cells as a single agent and in combination with Decitabine [44].

Other strategies investigated in AML include treatments blocking the PD-1/PD-L1 axis.

A phase 2 study showed that the association between the PD1 inhibitor nivolumab and AZA determines an encouraging response rate and overall survival in patients with R/R AML; the greatest results were seen in the subset of patients who had not been previously exposed to hypomethylating agents [45]. Similarly, Gojo et al. showed that the association of the PD1 inhibitor pembrolizumab with AZA led to promising results in terms of response rates in R/R AML patients, with greater clinical activity in newly diagnosed, older AML patients [46].

The association of the anti-PD-L1 human monoclonal antibody (mAb) Durvalumab with AZA led to unsatisfactory results with superimposable efficacy to AZA alone.

Monalizumab, a humanized immunoglobulin G4 (IgG4) mAb targeting the NK and T cell CI NKG2A, showed promising activity in AML murine models [47,48,49,50].

Sabatolimab is a humanized IgG4 mAb directed against the human T cell immunoglobulin domain and mucin domain-3 (TIM-3), an immune-regulatory receptor that is expressed on immune cells and leukemic blasts but not on hematopoietic stem cells. Sabatolimab is currently being investigated in the post-HSCT setting for MRD-positive AML patients, and several trials are investigating its association with HMA and HMA/venetoclax for AML patients ineligible for standard intensive chemotherapy.

Most recently, Cusatuzumab, an anti-CD70 mAb targeting the CD27-CD70 axis, has also been investigated in AML, mainly in combination with HMA agents. Initial results showed increased NK-cell-mediated killing and decreased leukemic blast cells in AML patients [51].

## 5. Target Antigens for Cell-Based Immunotherapy in AML

The efficacy of immune-based therapies relies on the identification of target molecules, which allow for the recognition and killing of blast cells while sparing healthy hematopoietic precursors or other tissues. Therefore, to maximize efficacy and reduce off-target toxicities, an ideal target should be strongly expressed on the surface of AML blasts with no or limited expression on healthy bone marrow or extra hematopoietic cells [52].

Unfortunately, leukemia-specific antigens consisting of tumor-specific proteins (i.e., resulting from gene mutations as mutated NPM1, FLT3-ITD and PML RARa), which would represent the ideal target for immunotherapy, are mainly expressed at the intracellular level and, therefore, are not suitable for immunotherapy strategies.

Several other antigens have already been explored and proposed as target for clinical use. Table 1 provides an overview of the most relevant target antigens for which the development of cell-based immunotherapy strategies is more advanced.

Lineage-specific antigens (i.e., CD33) are suitable targets; however, dose-limiting toxicities are often observed due to high cross-expression on hematopoietic precursors.

Leukemia-associated antigens could represent a useful target given their overexpression on AML blasts. Non-lineage specificity represents the main limitation in their application since they can be found on both healthy hematopoietic cells, resulting in hematologic toxicity, and non-hematopoietic tissues, with subsequent off-tumor toxicities.

### 5.1. CD33

CD33 is a myeloid-specific sialic-acid-binding receptor expressed on almost 90% of acute myeloid blasts and leukemic stem cells, thus representing a valid target for immunotherapy strategies. CD33 expression is downregulated in the late stages of myeloid development, resulting in low-level expression on peripheral granulocytes and tissue macrophages [53].

The antibody–drug conjugate, gemtuzumab-ozogamicin (GO), was developed and showed satisfactory results when added to conventional chemotherapy, in particular in the setting of favorable cytogenetics risk-AML. Based on randomized trials and on a meta-analysis of five trials involving more than 3000 AML patients, the association of GO with standard 3 + 7 induction was approved as a first-line treatment for adult patients diagnosed with CD33+ AML [54,55,56,57].

Very encouraging preclinical data have recently been published regarding improved antibody–drug conjugate and CD33-targeted strategies among these bispecific antibodies and chimeric antigen receptor T cells [58,59,60,61].

### 5.2. CD123

The interleukin-3 receptor alpha chain (IL-3Rα or CD123) is a cell membrane protein overexpressed in several hematologic malignancies; physiologically, it heterodimerizes with CD131 to form the active IL3 receptor complex. IL-3R is a member of the beta common (βc) family of receptors, which also includes IL-5R and the granulocyte-monocyte colony-stimulating factor (GM-CSF) receptor. This family of receptors regulates the growth, proliferation, survival and differentiation of hematopoietic cells, along with immunity and inflammatory responses [62].

CD123 is normally strongly expressed on plasmacytoid dendritic cells (PDC). Variable levels of expressions are reported in hematological malignancies, including AML [63], where CD123 expression has been associated with aggressive clinical behavior with enhanced blast proliferation and poor prognosis [64]. In addition to its variable expression rates on AML blasts, CD123 is a marker of leukemic stem cells (LSCs), representing an interesting target for immunological strategies aimed at leukemia eradication [65,66,67].

Several CD123-targeted strategies are in development, with some in advanced phases of clinical experimentation, including CD123-directed CAR-T cells and CD123 × CD3 bispecific T cell engagement, and are discussed in the specific paragraph [68,69,70,71].

### 5.3. CLL-1

The C-type lectin-like molecule-1 (CLL-1) belongs to group V of the C-type lectin-like receptor family, and plays a crucial role in the regulation of innate and adaptive immunity (anti-infectious responses and immunological homeostasis). CLL-1 is widely expressed on AML cells (almost 90%) and LSCs, while it is absent on granulocyte-macrophage progenitors. Due to these features, CLL-1 can be considered an ideal target for AML treatment [52,72,73]. CAR-T targeting cell therapy (CCL-1) in adults with Relapsing/Refractory (R/R) AML has been tested in clinical trials. In a recent trial, Jin X et al. reported the positive efficacy and tolerable safety of CLL-1 CAR-T cell therapy in adult R/R AML. It was tested in 10 patients with R/R AML, with 70% efficacy with full response or partial response with incomplete blood count recovery [74].

### 5.4. FLT3

The FMS-like tyrosine kinase 3 (FLT3/CD135) is a member of the class III receptor tyrosine kinases, sharing its homology with other members of the family, such as the platelet-derived growth factor receptor (PDGFR), stem cell factor receptor (KIT) and colony-stimulating factor 1 receptor (CSF1R). In healthy bone marrow, FLT3 is selectively expressed on the surface of hematopoietic stem cells and immature hematopoietic progenitors, playing an important role in the regulation of hematopoiesis, including proliferation, differentiation and survival [75].

Activating mutations in the FLT3 gene, including internal tandem duplications (ITDs) and missense point mutations in the tyrosine kinase domain (TKD), is one of the most common molecular abnormalities in AML. Genetic alterations of FLT3 lead to the overexpression or constitutive activation of the tyrosine kinase receptor in AML blast, with the subsequent survival and proliferation of AML cells.

Like CLL-1, FLT3 shows favorable expression on AML blasts (>90%) and LSCs, with low expression on healthy HSCs [52,76].

### 5.5. NKG2D

NKG2D is a transmembrane c-type lectin-like activating receptor expressed on immune-effector cells, mostly NK, stimulating cytotoxic activity. NKG2D ligands (NKG2DL) are commonly absent in healthy cells, being upregulated in response to infections and oncogenic transformation. NKG2DL comprises MHC class I chain-related protein A and B (MICA, MICB) and the UL16-binding proteins 1–6 (ULBP1-6). CAR-T and CAR-NK cells have been developed expressing the NKG2D ectodomain fused to cytoplasmic signaling domains. The recognition of the NKG2D ligandome is able to induce T and NK activation. Promising albeit heterogeneous results have been reported in AML and myelodysplastic syndromes [44,77].

These discrepancies in outcome could possibly be related to the epigenetic and post-translational mechanisms regulating NKG2DL, in addition to the lower affinity of NKG2D for their ligands. Moreover, in hepatocellular carcinoma, it has been shown that the existence of a soluble form of NKG2DL can mediate tumor immune escape through the blocking of the NKG2D signaling pathway [78].

Indeed, combining strategies that exploit epigenetic modifiers able to induce the upregulation of NKG2DLs could improve the efficacy of NKG2D-targeted immunological interventions [44].

## 6. Chimeric Antigen Receptor Cells

### 6.1. CAR-T

Since the 1980s, T cells modified with the lentiviral chimeric antigen receptor (CAR) gene have been developed. The chimeric antigen receptor is made of a monoclonal antibody capable of binding to specifically identified target antigens, coupled with the activation of the intracellular proliferation signal domain [79]. When the chimeric receptor binds to the antigen expressed in malignant cells, it stimulates the activation signal, leading to CAR-T cell activation and killing effects (Figure 1B). When designing a CAR-T cell, the choice antigen receptor is very important as the CAR target should be highly expressed on tumor cells and weak or absent on healthy cells. CAR-T cells against the CD19 protein have been shown to be effective against acute lymphoblastic leukemia, B cell lymphoma and lymphocytic leukemia, and have been approved by the FDA and EMA in those settings.

In the AML setting, designing a CAR-T is particularly challenging, as potential targets are often expressed on healthy myeloid cell compartments, resulting in unacceptable toxicity and myelosuppression. Despite the limitation of the potential usefulness in AML, many CAR-T cell adoptive cell transfer therapies have been tested in early phase clinical trials [80,81].

An overview of the most significant clinical trials involving CAR-T in AML is provided in Table 2.

### 6.2. CAR-NK

Given the potent anti-leukemic activity of NK cells and thanks to a better understanding of NK receptor functioning, a deep interest in exploiting anti-leukemic NK activity outside of the context of allogeneic HSCT has grown [82,83,84,85].

A pioneering study from Miller et al. showed that up to 1.5 × 10^7^/haploidentical NK cells/Kg could be safely infused in AML and cancer patients following Fludarabine/Cyclophosphamide (Flu/Cy) immunosuppressive light conditioning with evidence of clinical responses without GvHD [82].

Curti et al. reported on the feasibility and efficacy of adoptive immunotherapy with purified NK cells from a KIR-ligand mismatched haplo-identical donor in AML patients with R/R disease [85]. NK selection was performed using a two-step immunomagnetic separation for the purification of CD56+CD3- cells from the donor’s leukapheresis. NK cell infusion was performed after Flu/Cy immunosuppressive therapy and was followed by the subcutaneous administration of Interleukin-2. No specific NK-cell-related toxicities were observed. Donor-versus-recipient alloreactive NK cells were demonstrated in vivo by the detection of donor-derived NK clones and adoptively transferred NK cells were alloreactive against the recipient’s leukemic cells.

Subsequently, the same approach was extended to a cohort of elderly AML patients in first CR, not eligible for HSCT, as part of their consolidation therapy [86]. Interestingly, higher frequencies of donor NK alloreactive clones in the donors statistically correlated with better clinical outcomes, as well as the composition of NK grafts in terms of the frequency of alloreactive NK cells [86].

Albeit promising, these approaches have the limitation of requiring a haploidentical donor of NK cells. In order to overcome this issue, more recently, chimeric antigen receptor-modified NK cell therapy (CAR-NK) has been developed as a novel therapeutic option for hematological malignancies [87]. NK cells engineered with CAR expression maintain their capability of being stimulated by innate receptors, whilst their antigen recognition is redirected towards CAR-specific targets (Figure 1B). This feature maintains NK cell-mediated cytotoxicity against malignant cells, even in cases of target antigen downregulation. An advantage of NK-based immunotherapy is the fact that allogeneic NK cells do not induce GvHD, thus representing a readily available off-the-shelf therapy and overcoming some major limitations associated with autologous T cell manipulation, such as harvest and in vivo expansion.

Indeed, there are many NK cell resources that may provide good quality NK cells that are required for the engineering and development of CAR-NK cell therapy. These include: NK cell lines, peripheral blood NK cells and umbilical cord blood NKs, in addition to placenta and stem-cell-derived NK cells [88]. Moreover, the toxicity profile of CAR-NK favorably compares to T-lymphocyte-based immunotherapies for the negligible incidence of cytokine-release syndrome and neurotoxicity (see specific paragraph).

A recent study reported the generation of CD33-targeted CAR-modified NK cells from peripheral blood NK cells via baboon envelope pseudo-typed lentiviral vectors. Transduced cells showed a stable CAR-expression, continuous proliferation as well as increased cytotoxicity versus CD33-positive OCI-AML2 and primary AML cells in vitro. CAR-NK also prevented the bone marrow engraftment of leukemic cells in mice models with no notable adverse effects. An increased survival or improved BM homing of CAR-NK cells was also observed, with almost 80% of bone marrow and peripheral blood CAR-positive NK cells [89].

An overview of the most significant clinical trials involving CAR-NK in AML is provided in Table 3.

## 7. Strategies for T and NK Cells Engagement

### 7.1. BiTEs and DARTs

The term “bispecific antibodies” refers to a family of molecules specifically designed to simultaneously bind two different antigens located on immune effector cells and tumor cells. The idea of a hybrid antibody capable of activating effector T cells against target antigens dates back to 1985, and the CD3 polypeptides on T cells were identified to be the T cell engager component of the bispecific antibody [90,91].

Based on these data, the first research in this field focused on the creation of a bispecific antibody able to involve the cytotoxic activity of T cells with the development of molecular constructs, allowing the simultaneous binding of target antigens on the surface of malignant cells and the CD3 component of the T cell receptor complex (TCR) [92,93].

In the setting of hematological malignancies, the first bispecific T cell engager (BiTE) produced was blinatumomab, directed against CD3 on T cells and CD19 on blast cells, currently approved for the treatment of patients with relapsed/refractory B cell-precursor acute lymphoblastic leukemia (B-ALL) or patients with B-ALL with hematological complete remission but persistent minimal residual disease [94].

The great success achieved by blinatumomab set the basis for further exploration of bispecific antibodies in other hematological malignancies, such as AML, by targeting myeloid-specific antigens such as CD33, CD123 and CLL-1.

#### Mechanism and Structure

Bispecific antibodies are composed of a single heavy and light chain of the variable region of a tumor-associated antigen (TAA) and the CD3 of T cells, connected by a linker molecule.

The connection between T cells and target cells induces the formation of “immunologic synapses”, eliciting a cytotoxic response against the tumor cells via the CD3-transduced activating signaling cascade in T lymphocytes (Figure 1C) [95]. In their basic structure, BiTEs contain a linker sequence, the length of which determines the flexibility of the molecule and the antigen-binding kinetics [96,97].

Advances in bioengineering technologies have led to the creation of a new generation of bispecific antibodies. A variant construct of the bispecific engager is represented by dual affinity retargeting antibodies (DARTs), differing from BiTEs by the addition of a c-terminal disulfide bridge to the diabody backbone, which guarantees a better stabilization of the molecule (Figure 1C) [98].

Indeed, the downregulation of Major Histocompatibility Complex type 1 (MHC-1) and costimulatory molecules on tumor cells represent the main immunological escape mechanism towards T cells. However, bispecific antibodies show an ability to engage CD8 T cells independently from MHC-1 expression on tumor cells [92].

The major clinical limitation of first-generation bispecific antibodies consists of their short half-life, resulting in the need for a continuous infusion for administration; novel studies are focusing on the development of half-life-extending (HLE) bispecific antibodies, designed to be suitable for once-weekly dosing [60].

### 7.2. BiKEs and TRiKEs

A further evolution of immunotherapy is based on bispecific antibodies capable of engaging with NK cells. These include the development of Bi- and Tri-specific killer cell engagers, also known as BiKEs and TriKEs, which produce more efficient immunological synapses between NK cells and tumor cells.

These molecules share with BiTEs the backbone structure consisting of at least two single chain variable fragments (scFVs) connected by a linker sequence. The main difference consists of the engagement and activation of NK cells through the binding of CD16 by the scFV, while the other scFV remains specific to the tumor-associated target. In addition, TRiKEs, in their structure, present an additional portion consisting of an IL-15 crosslinker, which is able to expand the NK response. Anti-CD16 scFV BiKEs and TriKEs can further activate NK cells via CD16-Fc interaction by mediating the ADCC of opsonized target cells in view of the CD16 polymorphism and the presence of low-affinity CD16 allotypes. When compared to the use of mAbs, BiKEs and TriKEs show several advantages, such as increased biodistribution given their smaller composition, greater flexibility and indeed lower immunogenicity (Figure 1C). In addition, bispecific antibodies show the ability to engage CD8+ T cells independently from HLA-I expression on tumor cells. This represents a very important advantage, considering that the downregulation of HLA-I and costimulatory molecules on tumor cells represents one of the main tumor immune escape therapy mechanisms [99,100,101].

### 7.3. DARPins

Most recent strategies exploit the simultaneous targeting of multiple TAAs, in order to overcome immunotherapy limitations due to the heterogeneous expression of TAAs on AML blasts. In this way, DARPins (designed ankyrin repeat proteins) represent a novel class of small, single-domain, non-immunoglobulin proteins with multiple therapeutic potentials (Figure 1C). MP0533 is the first half-life extended avidity-engineered CD3 engaging DARPin able to simultaneously target CD33, CD123 and CD70. Preclinical studies have demonstrated that MP0533 is effective and able to induce T cell activation and the killing of AML blasts, with a better selectivity towards LSC over healthy hematopoietic stem cells (HSCs). Furthermore, with regard to safety, MP0533 has been proven to have a low risk of capillary leak syndrome (CLS) and a lower cytokine release syndrome (CRS) compared to mono-specific T cell engager approaches [102,103].

Given the promising preliminary results, a phase 1 clinical trial evaluating the safety and preliminary activity of MP0533 in humans is currently ongoing and recruiting (NCT05673057) [104].

### 7.4. ANKETs^TM^

ANKETs™ (Antibody-based NK cell Engagers) are multi-specific natural killer (NK) cell engagers that target specific TAAs on neoplastic cells (Figure 1C).

Preclinical studies have shown that the trifunctional NKp46-CD16a-NK engager (NKCE) targeting CD123 has good control of AML cell proliferation. Through its bond to NKp46, CD123-NKCE specifically targets NK cells and has potent antitumor activity against primary AML blasts, inducing NK cell activation and cytokine secretion. Moreover, in vivo studies have demonstrated strong overall survival in an aggressive human AML xenograft model [105].

These results supported the clinical development of CD123-NKCE (SAR443579). A phase 1/2 clinical trial evaluating this molecule is, at present, ongoing regarding patients with relapsed or refractory acute myeloid leukemia (R/R AML), B-cell acute lymphoblastic leukemia (B-ALL) or high-risk myelodysplastic syndrome (HR-MDS) [106].

An overview of the most significant clinical trials involving engager molecules is provided in Table 4.

## 8. New Toxicity Profile of Cell-Based Immunotherapy

The aim of cancer immunotherapy is to eradicate malignant cells by harnessing the power of the immune system. In this scenario, clinicians must face new specific therapy-related toxicities induced by the activation of immune-effectors. The most well-addressed side effect of novel immunological therapies is represented by cytokine release syndrome (CRS), consisting of a non-specific and abnormal activation of the immune system with the release of cytokines such as TNFα, IL-6 and IFNγ, and further activation of lymphocytes and myeloid cells.

The clinical manifestations and severity of immune system activation and cytokine release are variable from constitutional symptoms, including fever, malaise, myalgia, nausea, vomiting and headache to potentially life-threatening end-organ damage, including cardiovascular dysfunction, adult respiratory distress syndrome (ARDS), renal/hepatic failure and neurological manifestations.

The management of low-grade CRS consists of general supportive care measures, while grade ≥ 3 CRS generally requires immunosuppressive therapies, such as steroids and the anti-IL6 antibody tocilizumab. Vasopressors are required in case of hemodynamic instability unresponsive to fluid repletion [107].

Immune-effector cell-associated neurological syndrome (ICANS) is another possible toxicity of immunotherapy mostly associated with CAR-T infusion. Its clinical manifestations consist of progressive mental status changes, varying from mild symptoms such as confusion or disorientation to more severe cognitive impairment with agraphia and aphasia. ICANS can also occur in concomitance with CRS [108]. Its treatment mainly consists of steroids, in particular high doses of dexamethasone, eventually associated with tocilizumab in the case of CRS coexistence.

These emerging toxicities require close monitoring and intense support, especially in the cases of CRS or ICANS, requiring the prompt administration of immunosuppressive drugs and ICU transfer in critically ill patients.

## 9. Conclusions

Extended knowledge of AML biology and the mechanism of activity and regulation of immune cells has opened up the way to the development of novel, sophisticated strategies to exploit the immune system in anticancer treatment.

Cell-based immunotherapies can overcome mechanisms of chemo-resistance in AML and can be potentially exploited both in the setting of relapsed/refractory disease, as well as in early phases of treatment in association with standard regimens. Indeed, the association of novel immunological interventions could also allow for the development of reduced-intensity regimens for elderly and unfit patients.

The recent successes of CAR-T cells in the treatment of other hematological malignancies, which led to the approval of several products, have also paved the way for their use in the setting of AML. Unfortunately, different from B-cell malignancies, the heterogeneous nature of AML cells significantly complicates the development of targeted immunotherapy, and many products are still in the early development phase, with only a few advancing to further clinical studies.

From this perspective, the most recent CAR-T trials have investigated different, more specific targets, such as FLT3, and the preliminary results have shown comparable levels of anti-leukemic activity and lower off-tumor toxicity. Another limitation of CAR-T treatment is its complex logistics and that it requires a significant amount of time. To overcome these specific issues, albeit still in the very early stages of development, CAR-NK cell therapies represent an appealing, readily available off-the-shelf strategy.

Alternatively, bispecific and trispecific antibodies are novel agents with the ability to engage immune cells and targets through the simultaneous recognition of a tumor antigen and immune cell surface receptors. Owing to their logistical simplicity, several clinical trials have investigated their use in a clinical setting and the preliminary results are encouraging. The most promising targets are represented by CD33 and CD123, although their use may be limited by side effects, antigen escape and the potential nonselective activation of the immune system.

Larger amounts of data from clinical trials will permit the identification of the ideal setting and timing for the collocation of these novel approaches in the AML treatment setting.

## Figures and Tables

**Table 1:** Overview of the most relevant target antigens for which the development of cell-based immunotherapy strategies is more advanced.

Target Antigen	Type—Function	% of Expression	Potentialtarget Therapies
CD33	Lineage-specific Sialic acid-binding receptor—role as negative regulator of cell activation	Up to 90%	CAR-T, CAR-NK, BiTEs, BiKEs, TRiKEs, DARPin
CD123	Lineage-specific Alpha chain of the interleukin 3 receptor	Variable Higher in *NPM1*-mutated and *FLT3*-ITD AML	CAR-T, CAR-NK, DARTs, BiTEs, ANKET, DARPin
CLL-1	Leukemia-associated C-type lectin-like receptor—role in regulation of innate and adaptive immunity	90–95%	CAR-T, CAR-NK, BiTEs, TRiKEs,
(FLT3/CD135)	Leukemia-associated Class III receptor tyrosine kinases	>90%	CAR-T, BiTEs
NKG2DL	Not tumor-specific Ligand of C-type lectin-like receptor expressed on immune-effector cells-stimulates the cytotoxic activity	Upregulated in neoplastic cells	CAR-T, CAR-NK

**Table 2:** Overview of most significant clinical trials involving CAR-T in AML.

Product	Target	Patient Population	Phase	NCT
TAA05	FLT3 (CD135)	Relapsed/Refractory Acute Myeloid Leukemia	Early Phase I	NCT05445011
CI-135	FLT3 (CD135)	Relapsed/Refractory Acute Myeloid Leukemia	Early Phase I	NCT05266950
Anti-FLT3 CAR T-cells	FLT3 (CD135)	Relapsed/Refractory Acute Myeloid Leukemia	Early Phase I	NCT05023707
TAA-001	FLT3 (CD135)	Relapsed/Refractory Acute Myeloid Leukemia	Early Phase I	NCT05432401
Dual CD33/CLL1 CAR T Cells	CD33/CLL1	Relapsed/Refractory Acute Myeloid Leukemia	Phase I	NCT05248685
SC-DARIC33	CD33	Relapsed/Refractory Acute Myeloid Leukemia	Phase I	NCT05105152
CD33-CAR T cells	CD33	Relapsed/Refractory Acute Myeloid Leukemia	Phase I/II	NCT04835519
NKG2D-CAR-T cells	NKG2D	Acute Myeloid Leukemia; Myelodysplastic Syndrome; Multiple Myeloma	Phase I	NCT02203825
CD123-CAR T cells	CD123	Relapsed/Refractory Acute Myeloid Leukemia	Phase I	NCT04318678
UCART123	CD123	Relapsed/Refractory Acute Myeloid Leukemia	Phase I	NCT03190278
CD123-CAR T cells	CD123	Relapsed/Refractory Acute Myeloid Leukemia	Phase I	NCT04265963

**Table 3:** Overview of most significant clinical trials involving CAR-NK in AML.

Product	Target	Patient Population	Phase	NCT
CD33 CAR NK	CD33/CLL1	Relapsed/Refractory Acute Myeloid Leukemia	Early Phase I	NCT0521501
CD33/CLL1 CAR-NK	Anti-CD33	Relapsed/Refractory Acute Myeloid Leukemia	Phase I	NCT05008575
JD023	CD123	Relapsed/Refractory Acute Myeloid Leukemia	Early Phase I	NCT05574608
NKG2D CAR-NK	NKG2D	Relapsed/Refractory Acute Myeloid Leukemia	Phase I	NCT05247957
NKG2D CAR-NK	NKG2D	Relapsed/Refractory Acute Myeloid Leukemia	Phase I	NCT05734898

**Table 4:** Summary of principal clinical trials involving engager molecules in AML.

Product	NCT	Target	Patient Population	Phase	Outcome
AMV 564	NCT03144245	CD3 × CD33 bispecific antibody (BiTE)	Relapsed/Refractory Acute Myeloid Leukemia	Phase I	36 patients enrolled, median age 71 years Efficacy: Bone marrow blast reduction in 17 (49%) patients; 1 CR, 1 CRi, and 1 PR; 3 patients had hematologic improvement in neutrophil counts Safety: CRS ≥ grade 3: 0%, the most common Grade ≥ 3 AE has been anemia (11%) [58]
AMG 330	NCT02520427	CD3 × CD33 bispecific antibody (BiTE)	Relapsed/Refractory Acute Myeloid Leukemia	Phase I	55 patients enrolled, median age 58 years Efficacy: 3 CR (1 MRD negative), 4 Cri, 1 MLFS Safety: treatment-related AEs in 89%. CRS 60%; grade ≥ 3 in 7% [59].
AMG 673	NCT03224819	CD3 × CD33 bispecific antibody (BiTE-HLE)	Relapsed/Refractory Acute Myeloid Leukemia	Phase I	30 patients enrolled, median age 67.5 years Efficacy:Bone marrow blast reduction in 12/27 (44%) evaluable patients. 1 CRi. Safety: CRS in 50% (grade ≥ 3 in 4/15, 13%). Treatment-related serious AEs in 37% (grade ≥ 3 in 15/30, 50%; the most common were abnormal hepatic enzymes (17%), CRS (13%), leukopenia (13%), thrombocytopenia (7%), and febrile neutropenia (7%) [60].
APVO 436	NCT03647800	CD3 × CD123 bispecific antibody (BiTE)	Relapsed/Refractory Acute Myeloid Leukemia; Relapsed/Refractory Myelodysplastic Syndrome	Phase I/Ib	39 AML patients enrolled, median age 69 years. Efficacy: SD in 22/34 (64.7%) evaluable AML patients. Safety: treatment-related serious adverse events (SAEs) in 13/46 (28.3%) patients, the most common being CRS (7/13). Treatment-related transient neurotoxicity in 5/46 (10.9%) patients. CRS grade ≥ 3 in 4/46 (8.7%) patients, anemia grade ≥ 3 in 2/46 (4.3%) patients, infusion related reaction ≥ 3(IRR) in 2/46 (4.3%) patients [68].
Vibecotamab (XmAb14045)	NCT02730312	CD3 × CD123 bispecific antibody (BiTE)	Relapsed/Refractory Acute Myeloid Leukemia; B-cell Acute Lymphoblastic Leukemia; Blast Phase Chronic Myeloid Leukemia, Blastic Plasmacytoid Dendritic Cell Neoplasm	Phase I	104 AML patients enrolled, median age 63 years. Efficacy: ORR of 14% (2 CR, 3 Cri, 2 MLFS). SD in 36 (71%) patients. Safety: CRS in 62/106 (58.5%) patients, CRS grade ≥ 3 in 9/62 (15%) patients. IRR in 24% of patients [69].
Flotetuzumab (MGD006)	NCT02152956	CD3 × CD123 dual affinity retargeting antibody (DART)	Primary Induction Failure (PIF) and Early Relapse (ER) Acute Myeloid Leukemia	Phase I /II	38 PIF/ER AML patients enrolled, median age 63 yrs. Efficay: CRR for PIF pts was 45.8% (11/24; 5 CR, 3 CRh, and 3 CRi); CRR for ER pts was 35.7% (5/14; 2 CR, 1 CRh, 1 CRi and 1 MLFS). Safety: CRS was the most frequent AE, (grade ≤ 2). 9/38 (23.7%) pts experienced grade 1–2 headache; two pts experienced grade 3 confusion of short duration and fully reversable [70].
GEM333	NCT03516760	CD3 × CD33 bispecific antibody (BiTE)	Relapsed/Refractory Acute Myeloid Leukemia	Phase I	N/A
JNJ-67571244	NCT03915379	CD3 × CD33 bispecific antibody (BiTE)	Relapsed/Refractory Acute Myeloid Leukemia; high risk Myelodysplastic Syndrome	Phase I	N/A
JNJ-63709178	NCT02715011	CD3 × CD123 bispecific antibody (BiTE)	Relapsed/Refractory Acute Myeloid Leukemia	Phase I	62 patients enrolled, median age 67 years. Efficacy: no blast reduction observed. Safety: 63% total drug-related AEs (grade ≥ 3 in 84% of patients). CRS in 43.5% of patients. IRR in 11.3% of patients [71].
SAR440234	NCT03594955	CD3 × CD123 bispecific antibody (BiTE)	Relapsed/Refractory Acute Myeloid Leukemia; high risk Myelodysplastic Syndrome; B-cell Acute Lymphoblastic Leukemia	Phase I/II	7 patients enrolled, none enrolled in the Dose Expansion Part because of early termination of the study Efficacy: data not collected or analyzed due to the early termination of the study. Safety: 100% drug-related AEs (85.7% serious AEs). 28.6% CRS.
GTB-3550	NCT03214666	CD16/IL-15/CD33 Tri-Specific Killer Engager (TriKE^®^)	Relapsed/Refractory Acute Myeloid Leukemia; high risk Myelodysplastic Syndrome; Advanced Systemic Mastocytosis	Phase I/II	12 patients enrolled, 9 AML patients, 3 MDS patients. None enrolled in the Dose Expansion Part because of early termination of the study Efficacy:data not collected or analyzed due to the early termination of the study. Safety: 100% drug-related AEs (25% serious AEs). 16.7% CRS [61].
AMG427	NCT03541369	CD3 × FLT3 bispecific antibody (BiTE)	Relapsed/Refractory Acute Myeloid Leukemia	Phase I	N/A
CLN-049	NCT05143996	CD3 × FLT3 bispecific antibody (BiTE)	Relapsed/Refractory Acute Myeloid Leukemia; Myelodysplastic Syndrome	Phase I	N/A
MCLA-117	NCT03038230	CD3 × CLEC12A bispecific antibody (BiTE)	Relapsed/Refractory Acute Myeloid Leukemia; newly diagnosed elderly Acute Myeloid Leukemia	Phase I	N/A
SAR443579	NCT05086315	Trifunctional Natural Killer Cell Engager (NKCE) targeting the CD123 tumor antigen on cancer cells and co-engaging NKp46 and CD16a on NK cells	Relapsed/Refractory Acute Myeloid Leukemia; high risk Myelodysplastic Syndrome; B-cell Acute Lymphoblastic Leukemia	Phase I/II	N/A
MP0533	NCT05673057	Multispecific DARPin CD3 Engager Targeting CD33, CD123 and CD70	Relapsed/Refractory Acute Myeloid Leukemia; Myelodysplastic Syndrome	Phase I/II	N/A

## Data Availability

Data will be made available upon request to the corresponding author.
